# Construction of ceRNA Networks Associated With CD8 T Cells in Breast Cancer

**DOI:** 10.3389/fonc.2022.883197

**Published:** 2022-06-09

**Authors:** Zhilin Chen, Ruifa Feng, Ulf Dietrich Kahlert, Zhitong Chen, Luz Angela Torres-dela Roche, Amr Soliman, Chen Miao, Rudy Leon De Wilde, Wenjie Shi

**Affiliations:** ^1^ Department of Breast and Thoracic Oncological Surgery, The First Affiliated Hospital of Hainan Medical University, Haikou, China; ^2^ University Hospital for Gynecology, Pius-Hospital, University Medicine Oldenburg, Oldenburg, Germany; ^3^ Breast Center of The Second Affiliated Hospital of Guilin Medical University, Guilin, China; ^4^ Molecular and Experimental Surgery, University Clinic for General-, Visceral- and Vascular Surgery, University Medicine Magdeburg and Otto-von Guericke University, Magdeburg, Germany; ^5^ Department of Pathology, The First Affiliated Hospital of Nanjing Medical University, Nanjing, China

**Keywords:** ceRNA, T cell, breast cancer, lncRNA, target

## Abstract

**Background:**

The infiltration of CD8 T cells is usually linked to a favorable prognosis and may predict the therapeutic response of breast cancer patients to immunotherapy. The purpose of this research is to investigate the competing endogenous RNA (ceRNA) network correlated with the infiltration of CD8 T cells.

**Methods:**

Based on expression profiles, CD8 T cell abundances for each breast cancer (BC) patient were inferred using the bioinformatic method by immune markers and expression profiles. We were able to extract the differentially expressed RNAs (DEmRNAs, DEmiRNAs, and DElncRNAs) between low and high CD8 T-cell samples. The ceRNA network was constructed using Cytoscape. Machine learning models were built by lncRNAs to predict CD8 T-cell abundances. The lncRNAs were used to develop a prognostic model that could predict the survival rates of BC patients. The expression of selected lncRNA (XIST) was validated by quantitative real-time PCR (qRT-PCR).

**Results:**

A total of 1,599 DElncRNAs, 89 DEmiRNAs, and 1,794 DEmRNAs between high and low CD8 T-cell groups were obtained. Two ceRNA networks that have positive or negative correlations with CD8 T cells were built. Among the two ceRNA networks, nine lncRNAs (MIR29B2CHG, NEAT1, MALAT1, LINC00943, LINC01146, AC092718.4, AC005332.4, NORAD, and XIST) were selected for model construction. Among six prevalent machine learning models, artificial neural networks performed best, with an area under the curve (AUC) of 0.855. Patients from the high-risk category with BC had a lower survival rate compared to those from the low-risk group. The qRT-PCR results revealed significantly reduced XIST expression in normal breast samples, which was consistent with our integrated analysis.

**Conclusion:**

These results potentially provide insights into the ceRNA networks linked with T-cell infiltration and provide accurate models for T-cell prediction.

## Introduction

Breast cancer (BC) is by far the most frequently diagnosed kind of cancer and the fifth greatest cause of cancer-related deaths globally (1). About 2.3 million BC patients and 0.68 million deaths were reported in 2020 (1). BC is a very heterogeneous illness, consisting of several biological subgroups with distinct genetic features and therapeutic implications. In 2000, BC was first separated into the luminal, basal-like, HER2+, and normal breast-like subtypes (2). Each subtype has its own set of molecular markers, outcomes, clinical characteristics, and therapy responses. For example, the prognosis of patients with the luminal subtype is relatively good (3), and the primary treatments are mainly based on endocrine therapy (4). However, the prognosis of BC samples in the basal-like subtype is very poor (5), and there is a dearth of effective therapies available (6). Despite advancements in BC treatment, advanced or metastatic BC continues to have a dismal survival rate of 25% (7). Thus, novel therapeutic agents are needed.

Immunotherapy has been utilized to treat advanced breast cancer with metastases in the past few years. Immune-checkpoint inhibitors aim to block suppressive immune receptors and activate dysfunctional T cells, including CD8+ T cells. In a randomized, double-blind, and placebo-controlled clinical trial that contained 1,174 TNBC patients, the complete response rate was 63% (95% CI, 59.5%–66.4%) for patients in the group of pembrolizumab/chemotherapy compared with 56% (95% CI: 50.6%–60.6%) for the group of chemotherapy alone (8). The Food and Drug Administration authorized the use of pembrolizumab with chemotherapy for TNBC patients on July 26, 2021, based on these clinical study findings. This approval, however, brought up a new issue: how to distinguish between cancer patients who are immunotherapy sensitive and those who are immunotherapy insensitive. CD8 T cells are the most potent effectors in the anticancer immune response, and they are the foundation of cancer immunotherapy (9). Several indicators, especially CD8 T lymphocytes (TILs), have been identified to predict immunotherapy response (10). Thus, the prediction of CD8 T cells will contribute to the screening of immunotherapy-responsive BC patients.

On the other hand, lncRNAs are a novel class of ncRNAs that have more than 200 nucleotides in length. Dysregulation of lncRNAs has been implicated in the development of BC, involving cell growth, apoptosis, migration, and therapy resistance regulation (11). Salmena et al. proposed the competitive endogenous RNAs (ceRNAs) hypothesis (12) whereby RNA transcripts can communicate with each other *via* miRNA response elements (MREs). By competitively interacting with miRNAs, the lncRNAs can act as ceRNAs and positively regulate mRNAs. These ncRNAs act as ceRNAs to modulate mRNA expression and regulate protein levels, which contributes to the occurrence and development of tumors (13). Studies have shown that each miRNA can control the transcriptional expression levels of hundreds of proteins. Each mRNA contains different MREs, and so it may be targeted by multiple miRNAs (14). Numerous investigations have shown that ceRNAs could play critical roles in the genesis, progression, and outcome of BC (15). However, the ceRNA network that could influence the CD8 T cells has not been extensively studied so far.

Using bioinformatic analysis, our team discovered numerous lncRNA, miRNA, and mRNA, and constructed ceRNA networks associated with T cells. Machine learning models were constructed to predict the T cells by hub lncRNAs from the ceRNA networks. A risk score model with good prognostic predictive accuracy was constructed by hub lncRNAs from the ceRNA networks. These models may hold considerable promise for personalized therapy and prognosis prediction in BC patients.

## Materials and Methods

### Breast Cancer Dataset

The level 3 mRNA expression profiles of BC were obtained from The Cancer Genome Atlas (TCGA) by the “TCGAbiolinks” R package (16). A total of 1,072 BC samples were found with available lncRNA and mRNA expression profiles, and 1,066 BC samples were found with available miRNA expression profiles. The lncRNA and mRNA expression data were converted from “fragments per kilobase million” into “transcripts per kilobase million”. We gathered clinical data on 1,087 BC patients, including their overall survival and progression-free survival data.

### Estimation of Immune Cell Types

The single-sample Gene Set Enrichment Analysis (ssGSEA) model was used to calculate the amounts of immune cells and to convert the genomic data into the values of 28 different categories of human immune cells (17), which included the cells that were thought to be anti-tumor such as “activated CD4 T cell” and “activated CD8 T cell”. Several pro-tumor immunity cells, including “regulatory T cell” and “type-2 T-helper cell”, were also obtained. Two groups of BC patients were established according to the median value of “activated CD8 T cell”. The microenvironment cell populations counter (MCP-counter) method (18), which allows the accurate quantification of immune and stromal cells by genomic data, was used in this study. The ESTIMATE, a tool for predicting tumor purity and the presence of infiltrating stromal/immune cells in tumor tissues using gene expression data, was used (19).

### Screening of DElncRNAs, DEmiRNAs, and DEmRNAs Between Low and High CD8 T Cell Groups

The expression profiles of 8,633 lncRNAs were obtained using the annotation profile from the GENCODE program. After excluding 25% of lncRNAs having the lowest mean expression value, 6,475 lncRNAs were retained for further study. The expression profiles of 2,236 miRNAs were obtained by the miRNA annotation from the miRBaseVersions.db R package. After removing 25% of lncRNAs with the lowest mean expression value, 1,677 miRNAs were retained for further investigation. The expression profiles of 19,618 mRNAs were obtained by the mRNA annotation of the GRCh38 project. After excluding 25% of mRNAs with the lowest mean expression value, 14,714 mRNAs were retained for further study.

The differentially expressed RNAs (DElncRNAs, DEmiRNAs, and DEmRNAs) between two T-cell groups were calculated by the R package edgeR (20). DElncRNAs and DEmiRNAs were filtered using p-value of 0.05 and |log2fold change| > 0.3 as criteria. DEmRNAs were filtered using p-value of 0.05 and |log2fold change| > 0.5 as cutoffs. Upregulation mRNAs (UmRNAs), down-regulation mRNAs (DmRNAs), upregulation miRNAs (UmiRNAs), downregulation miRNAs (DmiRNAs), upregulation lncRNAs (UlncRNAs), and downregulation lncRNAs (DlncRNAs) were defined by their expression values in the high CD8 T-cell group.

### Constructing lncRNA–miRNA–mRNA ceRNA Networks and Enrichment Analysis

Based on ceRNA theory, we constructed lncRNA–miRNA–mRNA ceRNA networks by lncRNA–miRNA and miRNA–mRNA correlations. First, we downloaded all the lncRNA–miRNA and miRNA–mRNA interactions that were downloaded from StarBase (http://starbase.sysu.edu.cn/) (21). The first ceRNA network that has a positive correlation value with CD8 T cells was constructed by selecting ncRNA–miRNA and miRNA–mRNA interactions that contained UmRNAs, DmiRNAs, and UlncRNAs. The second ceRNA network that has a negative correlation value with CD8 T cells was constructed by selecting ncRNA–miRNA and miRNA–mRNA interactions that contained DmRNAs, UmiRNAs, and DlncRNAs. In this study, Cytoscape software was utilized to visualize the network.

Since functional enrichment analysis is crucial for interpreting high-throughput omics data in life science, Gene Ontology (GO) and Kyoto Encyclopedia of Genes and Genomes (KEGG) pathway enrichment analyses were performed in R using the function of clusterProfiler (p-value <0.05) (22). The lncRNAs were ranked by the number of ncRNA–miRNA interactions. The top 4 lncRNAs were selected from the first ceRNA network, and the top 5 lncRNAs were selected from the second ceRNA network. These nine lncRNAs were defined as the hub lncRNAs.

### Machine Learning Models for Prediction of CD8 T Cells

Using the expression profiles of nine hub lncRNAs, machine learning models were built for the prediction of CD8 T cells. BC patients were equally and randomly divided into training and testing samples. Different machine learning models, including decision tree (DT), gradient boosting machines (GBM), generalized linear models (GLM), artificial neural networks (ANN), random forest (RF), and support vector machine (SVM), were chosen to build models to predict the CD8 T-cell group by the R “Caret” package (23). The parameters for each machine learning model were determined by the fivefold cross-validation. In order to evaluate the robustness and prediction accuracy of constructed models in BC, the “ROCR” package was used to calculate the value of the area under the ROC curve (AUC).

### Cox Regression Model for Prediction of Prognosis

These nine hub lncRNAs were used to construct the Cox model. The coefficients for nine lncRNAs were obtained. The risk score of the samples was calculated by the coefficients and lncRNA expression profiles. Based on the median value, BC individuals were categorized into high- and low-risk classes. The correlation analysis of clinical information includes overall survival, age, gender, stage, and T, N, and M classifications in the TNM system. The log-rank test was used for the Kaplan–Meier curves of patient survival analyses.

### Real-Time Quantitative PCR

Total RNA was extracted from patients’ breast cancer at the First Affiliated Hospital of Nanjing Medical University using TRIzol reagent (Invitrogen, CA, USA). Isolated RNA was reverse transcribed into cDNA using HiScript II Q RT SuperMix for qPCR (Vazyme Biotech Co.,Ltd. Nanjing, China) following standard protocols. Real-time quantitative PCR (qPCR) was performed with synthetic primers and ChamQ SYBR qPCR Master Mix (Vazyme Biotech Co., Ltd. Nanjing, China) with a Quant Studio 5 Real-Time PCR Detection System (Thermo Fisher Scientific, MA, USA). The relative expression levels of XIST were calculated and quantified with the 2^−ΔΔCt^ method after normalization with the reference β-actin expression. All primers used are listed in **Table 1**.

**Table 1:** Sequences of primers for real-time quantitative polymerase chain reaction.

Gene	Sequence (5′ -> 3′)
Xist	
Forward	TGGATAGAGGACCCAAGCGA
Reverse	CAAGACTGGCCCAGGCATAA
β-actin	
Forward	AGGATTCCTATGTGGGCGAC
Reverse	ATAGCACAGCCTGGATAGCAA

## Results

### DElncRNAs, DEmiRNAs, and DEmRNA in Low and High CD8 T Cell Groups

**Figure 1:**
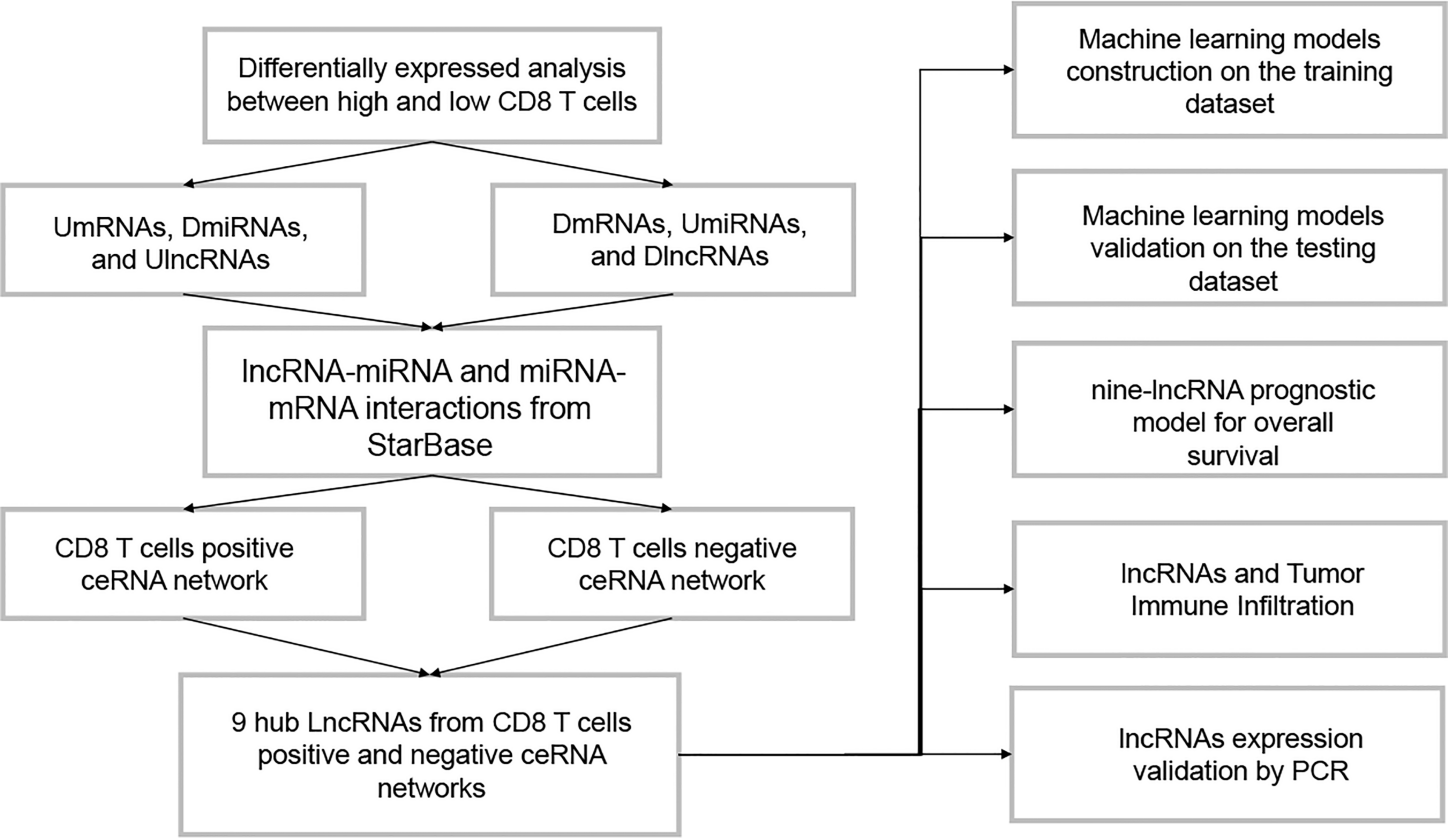
The flowchart of this study.

**Figure 2:**
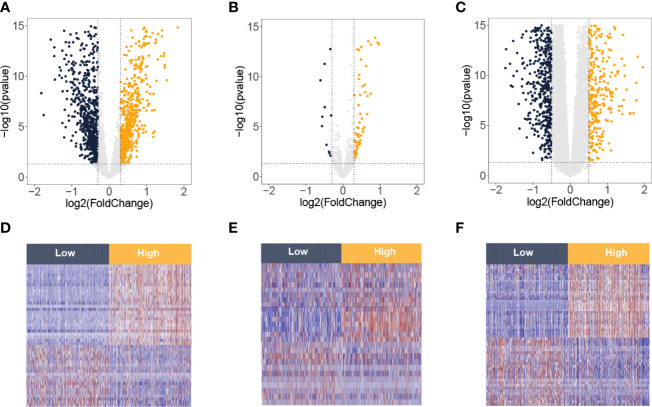
Differentially expressed lncRNA, miRNA, and mRNA between low and high T-cell groups from the TCGA-BRCA database. Volcano plots of differentially expressed lncRNAs **(A)**, miRNAs **(B)**, and mRNAs **(C)**. The red spots on the graphs reflect significantly elevated RNAs, whereas the green ones show significantly downregulated RNAs in the high T-cell group. Heatmaps of differentially expressed lncRNAs **(D)**, miRNAs **(E)**, and mRNAs **(F)**. The red signifies increased expression, whereas the blue signifies decreased expression.

The flowchart of this study is presented in **Figure 1**. By the ssGSEA and MCP-counter methods, values of CD8 T cells were calculated. High values of CD8 T cells were correlated with better BC prognosis (**Supplementary Figure 1**). Then, the BC samples were divided into two groups (low and high) by the median value of CD8 T cells from the ssGSEA algorithm. Based on the criteria of p-value <0.05 and |log_2_FC| > 0.3, 1,599 DElncRNAs (811 downregulation and 788 upregulation, **Figure 2A**) and 89 DEmiRNAs (11 downregulation and 78 upregulation, **Figure 2B**) between low and high CD8 T-cell groups were obtained. Based on the criteria of p-value <0.05 and |log_2_FC| > 0.5, 1,794 DEmRNAs (659 downregulation and 1,135 upregulation, **Figure 2C**). Heatmaps of the top DElncRNAs, DEmiRNAs, and DEmRNAs are presented in **Figures 2D–F**. Upregulation mRNAs (UmRNAs), downregulation mRNAs (DmRNAs), upregulation miRNAs (UmiRNAs), downregulation miRNAs (DmiRNAs), upregulation lncRNAs (UlncRNAs), and downregulation lncRNAs (DlncRNAs) were defined by their expression values in the high CD8 T-cell group.

### Construction of the ceRNA Network

**Figure 3:**
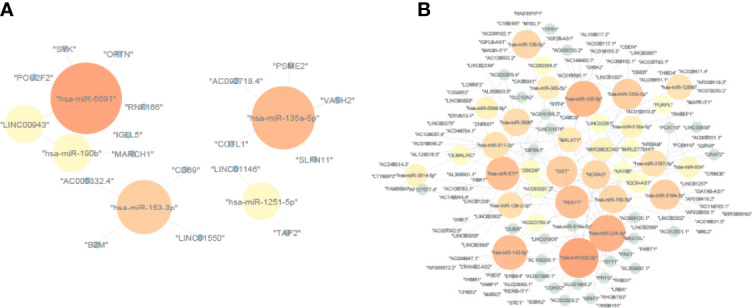
The ceRNA networks. **(A)** CD8 T-cell-positive ceRNA network constructed by UmRNAs, DmiRNAs, and UlncRNAs. **(B)** CD8 T-cell-negative ceRNA network constructed by DmRNAs, UmiRNAs, and DlncRNAs. The upregulation mRNAs (UmRNAs), downregulation mRNAs (DmRNAs), upregulation miRNAs (UmiRNAs), downregulation miRNAs (DmiRNAs), upregulation lncRNAs (UlncRNAs), and downregulation lncRNAs (DlncRNAs) were defined by their expression values in the high CD8 T-cell group.

We downloaded the lncRNAs–miRNAs and miRNAs–mRNAs interactions from the StarBase database. Due to the ceRNA theory, lncRNAs and mRNAs have a positive regulatory interaction, while miRNAs and mRNAs have a negative regulatory interaction. Then, lncRNA–miRNA and miRNA–mRNA interactions that contain UmRNAs, DmiRNAs, and UlncRNAs were used to construct a CD8 T-cell positive ceRNA network (**Figure 3A**). The lncRNA–miRNA and miRNA–mRNA interactions that contain DmRNAs, UmiRNAs, and DlncRNAs were used to construct a CD8 T-cell negative ceRNA network (**Figure 3B**).

### Gene Set Enrichment Analysis

To outline the potential function of the genes in the ceRNA networks, the pathways from the CD8 T-cell positive ceRNA network and the CD8 T-cell negative ceRNA network were obtained. Top GO terms and KEGG pathways in the CD8 T-cell positive ceRNA network included many immune pathways such as “antigen processing and presentation” (**Supplementary Table 1**). Top GO terms and KEGG pathways in the CD8 T-cell negative ceRNA network contained many cancer pathways such as “proteoglycans in cancer” (**Supplementary Table 2**).

### Machine Learning Models for the Prediction of CD8 T Cell Groups

**Figure 4:**
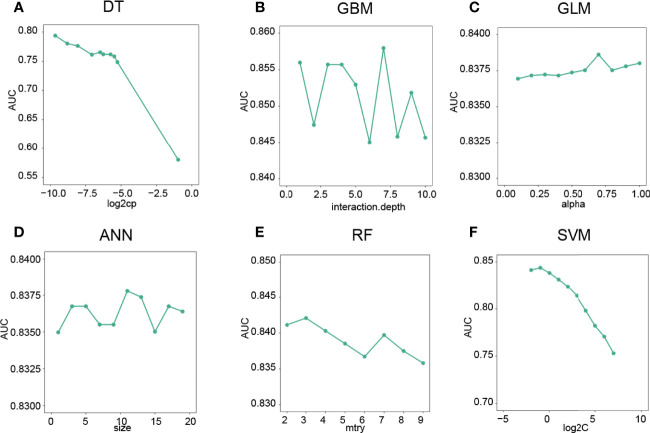
Optimal parameter selection for machine learning models. **(A)** The “log2CP” and corresponding AUC values of the DT algorithm. **(B)** The “interaction depth value” and corresponding AUC values of the GBM algorithm. **(C)** The “alpha value” and corresponding AUC values of the GLM algorithm. **(D)** The “Size” and corresponding AUC values of the ANN algorithm. **(E)** The mtry’ and corresponding AUC values of the RF algorithm. **(F)** The “log2C” value and corresponding AUC values of the SVM algorithm.

**Figure 5:**
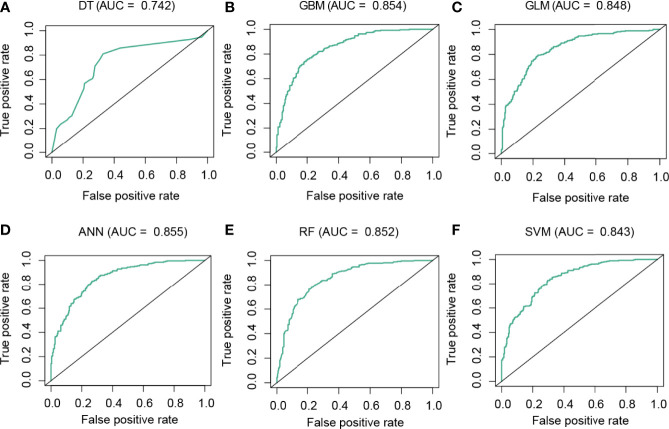
Performance analysis of the models in the test dataset. ROC curve analysis of the performance of DT **(A)**, GBM **(B)**, GLM **(C)**, ANN **(D)**, RF **(E)**, and SVM **(F)**.

To predict the CD8 T-cell group of BC samples, we constructed a model based on the hub lncRNAs from the CD8 T-cell-positive/negative ceRNA networks. The top 4 lncRNAs from the CD8 T-cell-positive ceRNA network, and the top 5 lncRNAs from the CD8 T-cell-negative ceRNA network were selected. These nine lncRNAs (NEAT1, XIST, NORAD, MALAT1, MIR29B2CHG, LINC00943, AC005332.4, AC092718.4, and LINC01146) were defined as the hub lncRNAs, and the expression profiles of these hub lncRNAs were kept for further analysis. The expression profiles were evenly and randomly split into a training (50%) and a testing set (50%). Fivefold cross-validation was conducted on the training set to select the best parameters for DT, GBM, GLM, ANN, RF, and SVM. The models were trained on the training set with the selected best parameters. The log2CP value from DT was selected as “−9.65”, since it achieved the highest AUC value of 0.794 in the fivefold cross-validation (**Figure 4A**). The interaction depth value from GBM was selected as “7”, since it achieved the highest AUC value of 0.857 in the fivefold cross-validation (**Figure 4B**). The alpha value from GLM was selected as “0.7”, since it achieved the highest AUC value of 0.838 in the fivefold cross-validation (**Figure 4C**). The size value from ANN was selected as “11”, since it achieved the highest AUC value of 0.837 in the fivefold cross-validation (**Figure 4D**). The mtry value from RF was selected as “3”, since it achieved the highest AUC value of 0.842 in the fivefold cross-validation (**Figure 4E**). The log2C value from SVM was selected as “−1”, since it achieved the highest AUC value of 0.843 in the fivefold cross-validation (**Figure 4F**). Prediction accuracy was then measured by the AUC value calculated on the test set. On the testing set, the AUC values for DT, GBM, GLM, ANN, RF, and SVM were 0.742, 0.854, 0.848, 0.855, 0.852, and 0.843 (**Figures 5A–F**). The calculation process of the final model for DT is presented in **Supplementary Figure 2**.

### Establishment of the lncRNA Prognostic Model

**Figure 6:**
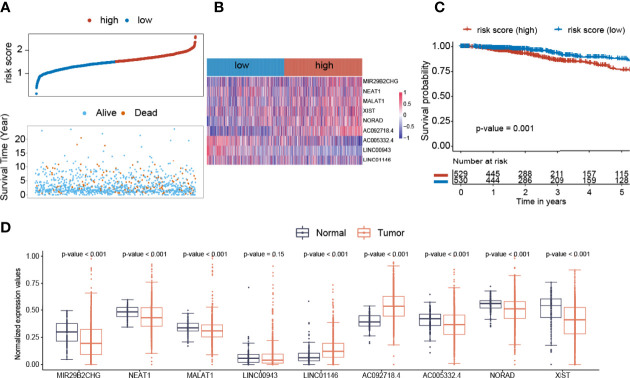
Construction of the Cox regression model. **(A)** The distribution of prognosis including survival status and survival time between two risk score groups. **(B)** The heatmap of nine lncRNAs expression profiles. **(C)** The overall survival analysis of risk score groups. **(D)** The expression distribution values of nine lncRNAs between normal and breast cancer samples.

Thereafter, we developed a nine-lncRNA prognostic model for overall survival prediction of BC patients. The risk score = (0.61 * MIR29B2CHG) + (0.027 * NEAT1) + (0.006 * MALAT1) + (−1.21 * LINC00943) + (0.24 * LINC01146) + (2.1 * AC092718.4) + (−1.02 * AC005332.4) + (0.99 * NORAD) + (0.193 * XIST). The distribution of prognosis, including survival time and status in risk score groups, is presented (**Figure 6A**). Additionally, the increased expression of AC005332.4 and LINC00943 was observed in the low-risk BC samples. On the other hand, MIR29B2CHG, MALAT1, XIST, NORAD, and AC092718.4 expression levels were greater in the high-risk BC samples (**Figure 6B**). Low-risk individuals in the BC cohort survived longer than high-risk ones when it came to OS (p-value = 0.001, **Figure 6C**). MIR29B2CHG, NEAT1, MALAT1, AC005332.4, NORAD, and XIST were elevated in normal breast samples than in breast cancer samples (**Figure 6D**). LINC01146 and AC092718.4 were more elevated in breast cancer samples than in normal breast samples (**Figure 6D**).

### Relationship Between Hub lncRNAs and Tumor Immune Infiltration

**Figure 7:**
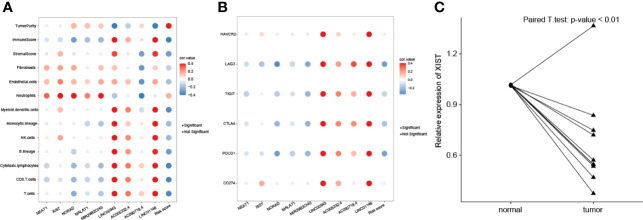
Correlations of lncRNA expression values and immune biomarkers. **(A)** Significant correlations between proportions of immune cells with nine lncRNAs expression profiles. **(B)** Significant correlations between levels of immune checkpoint genes with nine lncRNAs expression profiles. **(C)** The XIST relative expression levels of normal and breast cancer samples were compared.

We analyzed the associations among nine lncRNAs (NEAT1, XIST, NORAD, MALAT1, MIR29B2CHG, LINC00943, AC005332.4, AC092718.4, and LINC01146), risk score, tumor purity, and immune cell infiltration (**Figure 7A**). NEAT1, XIST, NORAD, MALAT1, and MIR29B2CHG were significantly negatively related to CD8 T cells, cytotoxic lymphocytes, and T cells. These five lncRNAs were significantly positively associated with fibroblasts, endothelial cells, and neutrophils. On the other hand, LINC00943, AC005332.4, AC092718.4, and LINC01146 were significantly positively associated with most types of immune cells, including T and B cells. Risk score value was found to be negatively related to immune cells but positively associated with tumor purity. NEAT1, XIST, NORAD, MALAT1, MIR29B2CHG, and risk value were significantly negatively related to immune checkpoint genes including LAG3, TIGIT, CTLA4, and PDCD1 (**Figure 7B**). LINC00943, AC005332.4, AC092718.4, and LINC01146 were significantly positively associated with LAG3, TIGIT, CTLA4, and PDCD1.

### Expression Patterns of Nine lncRNAs

Among nine lncRNAs, MIR29B2CHG, NEAT1, MALAT1, NORAD, and XIST were elevated in the low CD8 T-cell group than in the high CD8 T-cell group (**Supplementary Figure 3**). LINC00943, LINC01146, AC005332.4, and AC092718.4 were elevated in high CD8 T cells group than in low CD8 T cells group (**Supplementary Figure 3**). The associations between the risk model and clinical features were explored. Clinical characteristics, such as age, stage, T stage, N stage, and M stage, had no effect on the risk score (**Supplementary Figures 4A–E**). We found that a higher risk score was correlated with a negative prognosis on progression-free survival (**Supplementary Figure 4F**). Based on the best cutoff values of these nine lncRNAs, the samples were divided into high and low groups. The survival results showed that AC005332.4, AC092718.4, NORAD, and LINC00943 have significant correlations with prognosis (**Supplementary Figure 5**).

### Validation of XIST Expression in Breast Cancer Tissues by RT-qPCR

To validate XIST expression in BC clinical samples, 10 paired BC and normal breast tissues were collected and compared using real-time quantitative PCR (RT-qPCR). As shown in **Figure 7C**, expression of XIST is significantly lower in BC samples compared to the adjacent normal breast tissues (p-value <0.05).

## Discussion

Breast cancer is a major cause of mortality in the current world population (24). CD8 T cells have been considered to play a central role in immunotherapy (25). Breast cancer immunotherapy is gaining traction as a treatment option, especially for patients with metastatic disease (26). Thus, the prediction of CD8 T cells in the tumor microenvironment (TME) has broad significance for patients and clinicians. Apart from that, the lncRNA-based risk model is gaining popularity as a consequence of its greater predictive potential than other risk models. However, a detailed analysis of a predictive model based on lncRNAs that are associated with T cells has not yet been completed.

It is important to discriminate between immunotherapy-sensitive and immunotherapy-insensitive cancer patients. Several biomarkers for immunotherapy have been identified (10). For example, programmed death-ligand 1 (PD-L1)/programmed cell death-1 (PD-1) levels, tumor mutational burden (TMB), and CD8 T cells can be used to assess the efficacy of immunotherapy. However, since PD-L1 is a changing indicator, there is no established technique for its measurement (27). TMB detection is difficult, costly, and time consuming (28). The deficiency of T cell in TME was the primary factor contributing to immunotherapy tolerance (29). Thus, the prediction or detection of T-cell infiltration within tumors is a promising method for selecting immunotherapy patients.

We began our study by collecting differentially expressed lncRNAs, miRNAs, and mRNAs using differential analysis. Next, we built two ceRNA networks that had positive and negative correlations with CD8 T cells. Among the lncRNAs from two networks, nine lncRNAs were screened to construct a random forest model to predict the CD8 T cells, since these lncRNAs have more interactions with other miRNAs. The random forest model demonstrated excellent prediction ability in discriminating between high and low CD8 T cells, which might serve as a useful reference for personalized immunotherapy.

COX regression was used to construct the prognostic risk model. The value of MIR29B2CHG (C1orf132) was significantly attenuated in BC (30). NEAT1 expression was elevated in BC samples, and the increased expression of NEAT1 was associated with a poor overall survival in BC patients (31). MALAT1 levels were shown to be significantly related to breast cancer development and invasive capacity, indicating that MALAT1 acts as a metastasis suppressor (32). LINC00944 expression has a strong relationship with immune signaling pathways (33). There is not much research describing the roles of LINC00943, LINC01146, AC092718.4, and AC005332.4 in breast cancer. NORAD expression was considerably decreased in BC samples, and its deficiency was linked with poor tumor tissue development (34). XIST expression is significantly reduced in BC samples (35).

This study has some limitations. First, the CD8 T-cell values were predicted by the expression profiles and ssGSEA method. The CD8 T-cell values detected by experiments will increase the credibility of our study. Second, the prognostic values of these lncRNAs should be validated by an independent dataset. Third, the constructed model for immunotherapy response rate prediction should be tested by an independent dataset that contains the BC patients treated by immunotherapy.

## Conclusion

In summary, using miRNA/lncRNA/mRNA expression profiles in BC, we constructed two ceRNA networks that are correlated with CD8 T cells. Additionally, we found a combination of nine lncRNAs that may constitute models for CD8 T cells and prognosis prediction. This will serve as a foundation for future research on immunotherapy selection in BC patients.

## Data Availability Statement

The datasets presented in this study can be found in online repositories. The names of the repository/repositories and accession number(s) can be found in the article/**Supplementary Material**.

## Ethics Statement

The study was conducted according to the guidelines of the Declaration of Helsinki and approved by the Ethics Committee of Hainan Medical University (HYLL-2021-265). The patients/participants provided their written informed consent to participate in this study.

## Author Contributions

ZlC, UK, and RF: conceptualization, data curation, formal analysis, roles/writing—original draft, and writing—review and editing. ZtC, LR, and AS: roles/writing—original draft. CM, RW, and WS: funding acquisition, methodology, project administration, resources, and supervision.

## Conflict of Interest

The authors declare that the research was conducted in the absence of any commercial or financial relationships that could be construed as a potential conflict of interest.

## Publisher’s Note

All claims expressed in this article are solely those of the authors and do not necessarily represent those of their affiliated organizations, or those of the publisher, the editors and the reviewers. Any product that may be evaluated in this article, or claim that may be made by its manufacturer, is not guaranteed or endorsed by the publisher.

## Supplementary Material

Supplementary Figure 1 Overall survival analysis of activated CD8 T cells calculated by ssGSEA **(A)** and MCP-counter **(B)** in BRCA patients.

Supplementary Figure 2 The calculation process of the decision tree model.

Supplementary Figure 3 The expression distribution values of 9 lncRNAs between high and low CD8 T cell groups.

Supplementary Figure 4 The correlation of risk score with clinical parameters including age **(A)**, stage **(B)**, T **(C)**, N **(D)**, M **(E)**, and **(F)** progression-free survival.

Supplementary Figure 5 The survival analysis of 9 lncRNAs.
