# Supplementary figures and images for: Construction of ceRNA Networks Associated With CD8 T Cells in Breast Cancer
Source: Front Oncol. 2022 Jun 9;12:883197. doi: 10.3389/fonc.2022.883197 (PMC9219915; doi:10.3389/fonc.2022.883197)

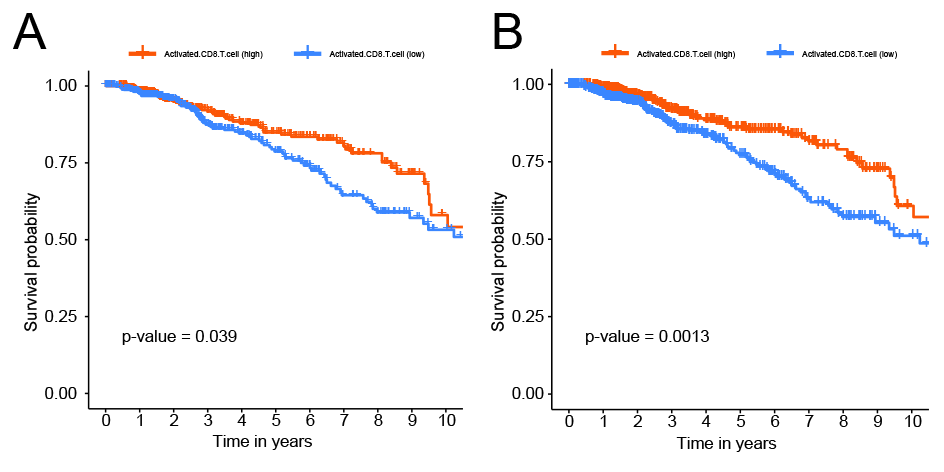

Supplement: Supplementary Figure 1 — Overall survival analysis of activated CD8 T cells calculated by ssGSEA (A) and MCP-counter (B) in BRCA patients. [file Image_1.tif]

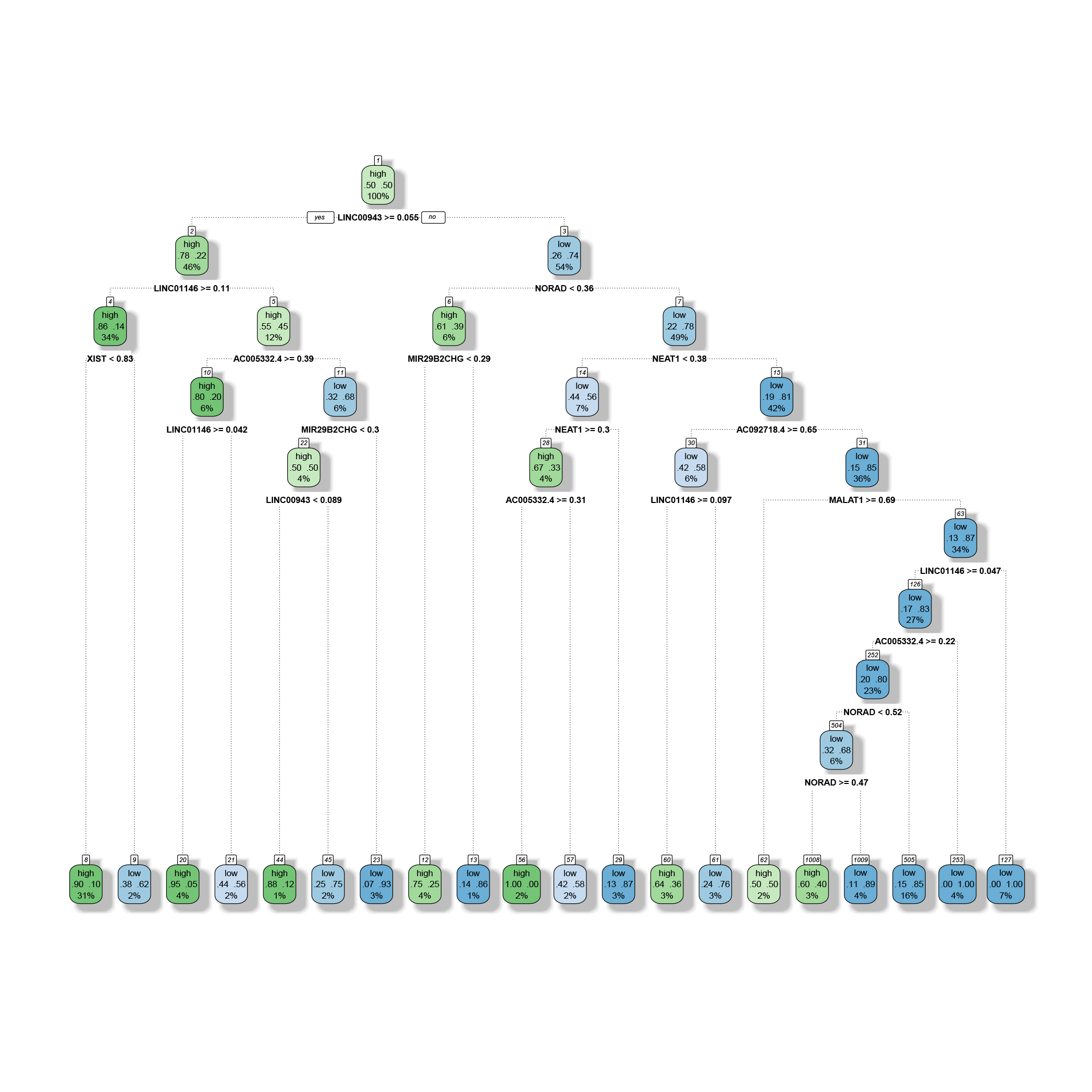

Supplement: Supplementary Figure 2 — The calculation process of the decision tree model. [file Image_2.tif]

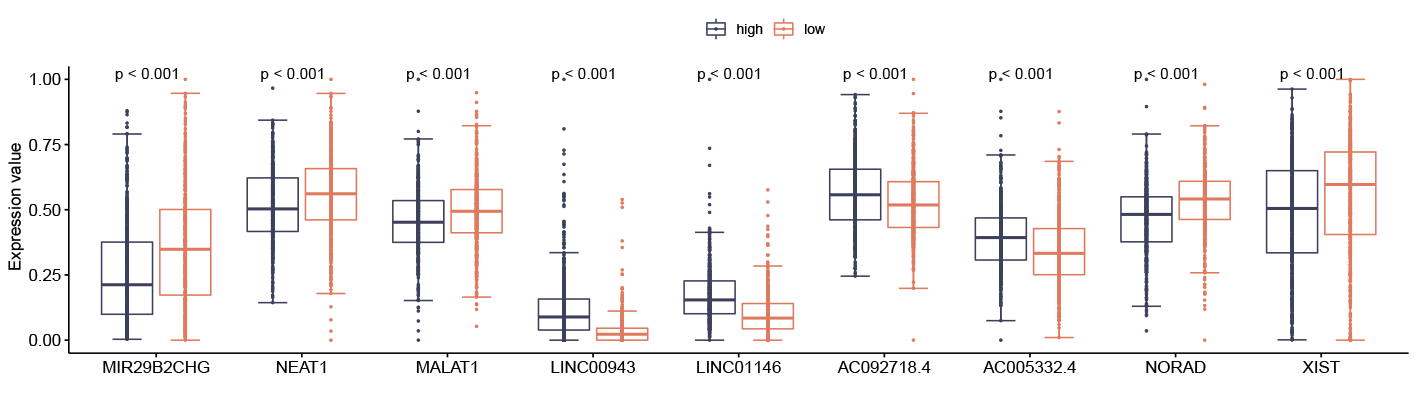

Supplement: Supplementary Figure 3 — The expression distribution values of 9 lncRNAs between high and low CD8 T cell groups. [file Image_3.tif]

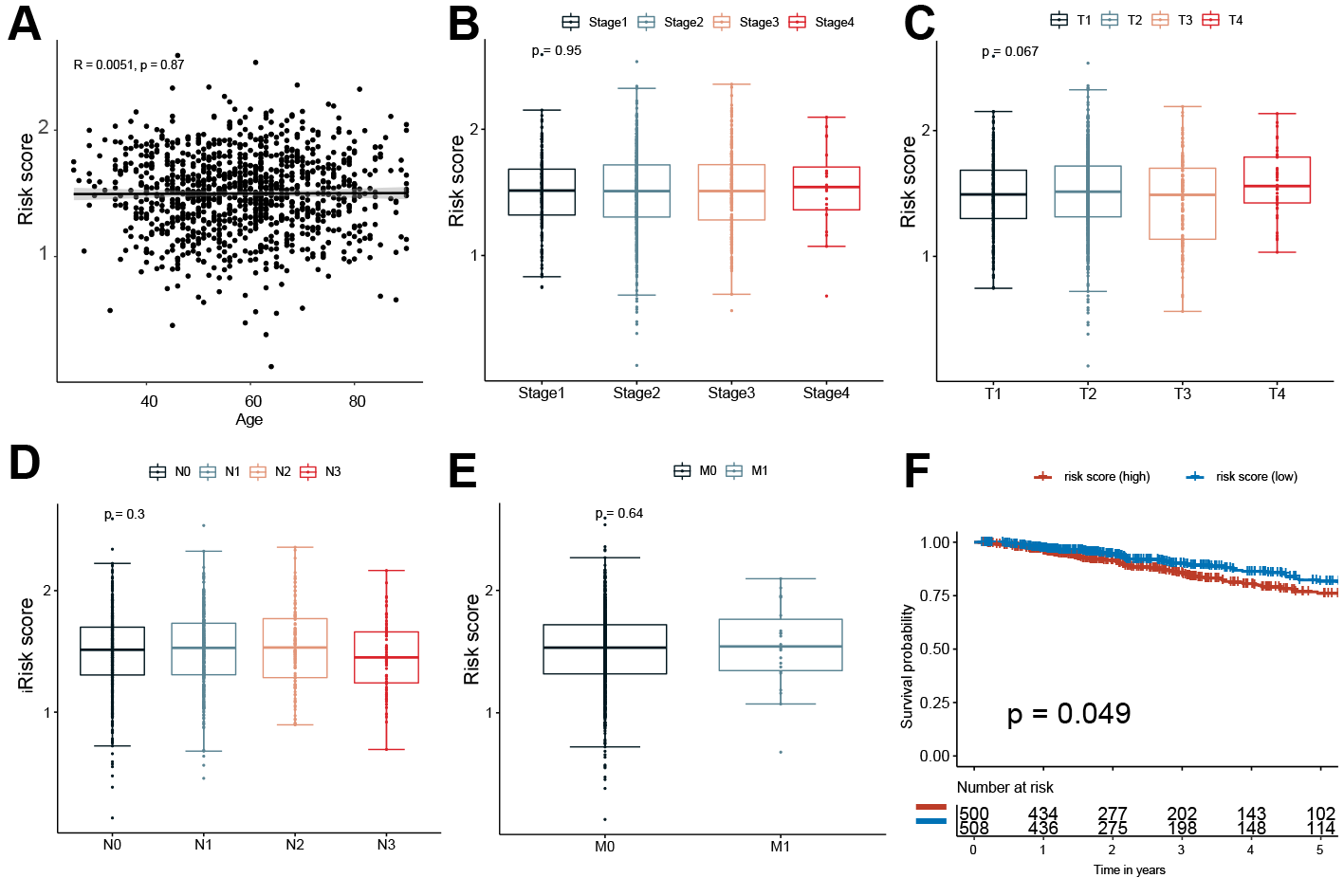

Supplement: Supplementary Figure 4 — The correlation of risk score with clinical parameters including age (A), stage (B), T (C), N (D), M (E), and (F) progression-free survival. [file Image_4.tif]

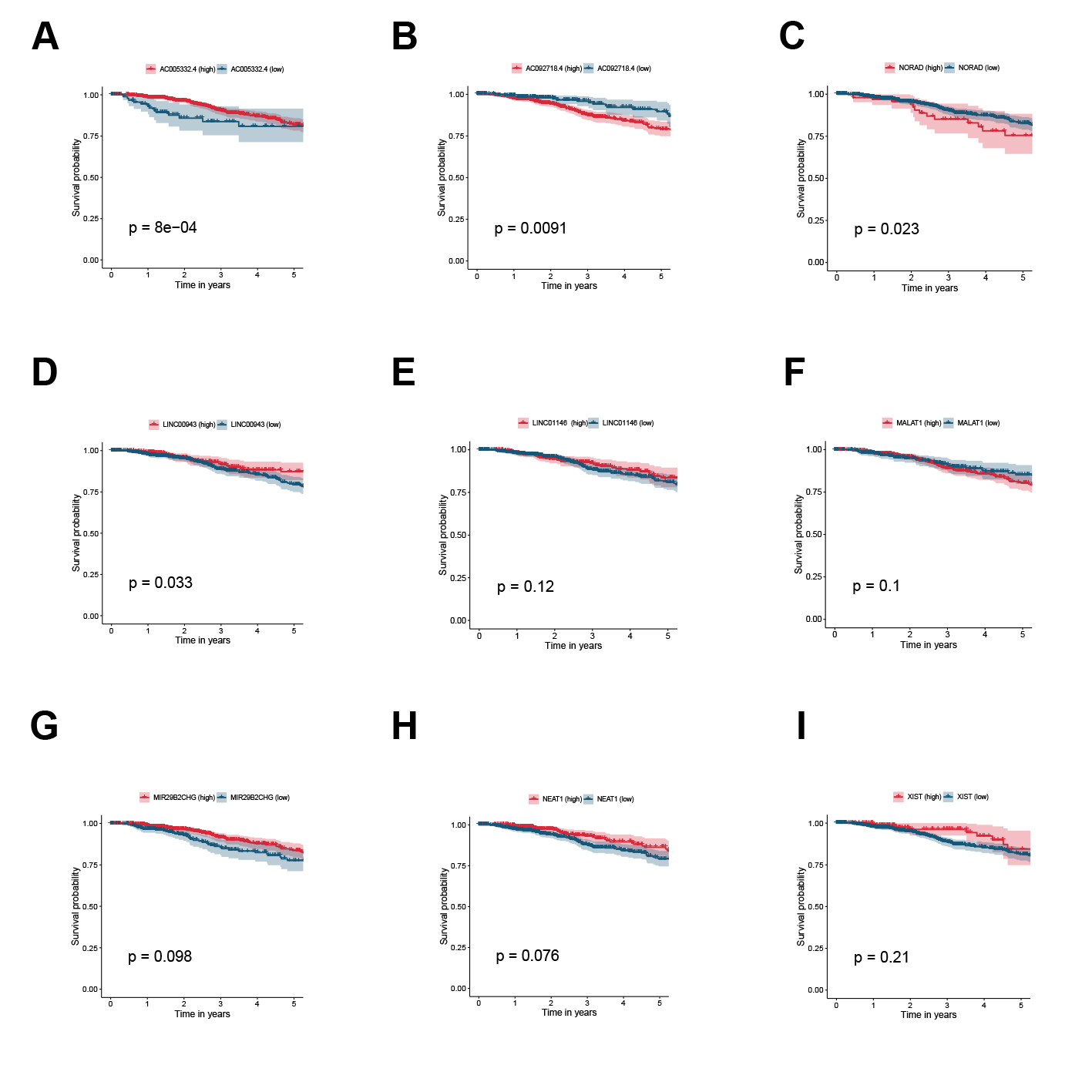

Supplement: Supplementary Figure 5 — The survival analysis of 9 lncRNAs. [file Image_5.tif]
