# Supplementary material for: Construction of ceRNA Networks Associated With CD8 T Cells in Breast Cancer
Source: Front Oncol. 2022 Jun 9;12:883197. doi: 10.3389/fonc.2022.883197 (PMC9219915; doi:10.3389/fonc.2022.883197)
Supplement: Supplementary file 6 [file DataSheet_1.docx]

| ID | Description | pvalue | Count | Type |
| --- | --- | --- | --- | --- |
| GO:0045335 | phagocytic vesicle | <0.01 | 3 | CC |
| GO:0030139 | endocytic vesicle | <0.01 | 3 | CC |
| GO:0030670 | phagocytic vesicle membrane | <0.01 | 2 | CC |
| GO:0055038 | recycling endosome membrane | <0.01 | 2 | CC |
| GO:0009897 | external side of plasma membrane | <0.01 | 3 | CC |
| GO:0140534 | endoplasmic reticulum protein-containing complex | <0.01 | 2 | CC |
| GO:0030176 | integral component of endoplasmic reticulum membrane | <0.01 | 2 | CC |
| GO:0030666 | endocytic vesicle membrane | <0.01 | 2 | CC |
| GO:0031227 | intrinsic component of endoplasmic reticulum membrane | <0.01 | 2 | CC |
| GO:0019814 | immunoglobulin complex | <0.01 | 2 | CC |
| GO:0002479 | antigen processing and presentation of exogenous peptide antigen via MHC class I, TAP-dependent | <0.01 | 3 | BP |
| GO:0042590 | antigen processing and presentation of exogenous peptide antigen via MHC class I | <0.01 | 3 | BP |
| GO:0002474 | antigen processing and presentation of peptide antigen via MHC class I | <0.01 | 3 | BP |
| GO:0002475 | antigen processing and presentation via MHC class Ib | <0.01 | 2 | BP |
| GO:0019885 | antigen processing and presentation of endogenous peptide antigen via MHC class I | <0.01 | 2 | BP |
| GO:0002483 | antigen processing and presentation of endogenous peptide antigen | <0.01 | 2 | BP |
| GO:0019883 | antigen processing and presentation of endogenous antigen | <0.01 | 2 | BP |
| GO:0002478 | antigen processing and presentation of exogenous peptide antigen | <0.01 | 3 | BP |
| GO:0019884 | antigen processing and presentation of exogenous antigen | <0.01 | 3 | BP |
| GO:0048002 | antigen processing and presentation of peptide antigen | <0.01 | 3 | BP |
| hsa04612 | Antigen processing and presentation | <0.01 | 3 | KEGG |
| hsa05168 | Herpes simplex virus 1 infection | <0.01 | 4 | KEGG |
| hsa05169 | Epstein-Barr virus infection | <0.01 | 3 | KEGG |
| hsa05170 | Human immunodeficiency virus 1 infection | 0.01 | 2 | KEGG |
| hsa05163 | Human cytomegalovirus infection | 0.01 | 2 | KEGG |
| hsa05340 | Primary immunodeficiency | 0.02 | 1 | KEGG |
| hsa02010 | ABC transporters | 0.03 | 1 | KEGG |
| hsa03050 | Proteasome | 0.03 | 1 | KEGG |
| hsa04664 | Fc epsilon RI signaling pathway | 0.04 | 1 | KEGG |

**Supplementary Table 1.** Top GO terms and KEGG pathways in CD8+ T cells positive ceRNA network were analyzed.

| ID | Description | pvalue | Count | Type |
| --- | --- | --- | --- | --- |
| GO:0045197 | establishment or maintenance of epithelial cell apical/basal polarity | 0.000125898 | 3 | BP |
| GO:0007163 | establishment or maintenance of cell polarity | 0.000161215 | 5 | BP |
| GO:0035088 | establishment or maintenance of apical/basal cell polarity | 0.000179283 | 3 | BP |
| GO:0061245 | establishment or maintenance of bipolar cell polarity | 0.000179283 | 3 | BP |
| GO:0060560 | developmental growth involved in morphogenesis | 0.000212627 | 5 | BP |
| GO:0098984 | neuron to neuron synapse | 1.22E-05 | 7 | CC |
| GO:0014069 | postsynaptic density | 7.80E-05 | 6 | CC |
| GO:0032279 | asymmetric synapse | 8.51E-05 | 6 | CC |
| GO:0099572 | postsynaptic specialization | 0.000109539 | 6 | CC |
| hsa04144 | Endocytosis | 4.06E-05 | 6 | KEGG |
| hsa05205 | Proteoglycans in cancer | 0.00018191 | 5 | KEGG |
| hsa04730 | Long-term depression | 0.000535869 | 3 | KEGG |
| hsa04114 | Oocyte meiosis | 0.005061952 | 3 | KEGG |
| hsa04720 | Long-term potentiation | 0.013952484 | 2 | KEGG |
| hsa04012 | ErbB signaling pathway | 0.021876713 | 2 | KEGG |
| hsa04970 | Salivary secretion | 0.025876034 | 2 | KEGG |
| hsa04713 | Circadian entrainment | 0.027979736 | 2 | KEGG |
| hsa04914 | Progesterone-mediated oocyte maturation | 0.030703745 | 2 | KEGG |
| hsa04972 | Pancreatic secretion | 0.030703745 | 2 | KEGG |

**Supplementary Table 2.** Top GO terms and KEGG pathways in CD8+ T cells negative ceRNA network were analyzed.
